# Targeting Aerobic Glycolysis and HIF-1α Expression Enhance Imiquimod-induced Apoptosis in Cancer Cells

**DOI:** 10.18632/oncotarget.1734

**Published:** 2014-03-12

**Authors:** Shi-Wei Huang, Jun-Kai Kao, Chun-Ying Wu, Sin-Ting Wang, Hsin-Chen Lee, Shu-Mei Liang, Yi-Ju Chen, Jeng-Jer Shieh

**Affiliations:** ^1^ Institute of Biomedical Sciences, National Chung Hsing University, Taichung, Taiwan; ^2^ Department of Pediatrics, Children's Hospital, Changhua Christian Hospital, Changhua, Taiwan; ^3^ Division of Gastroenterology and Hepatology, Taichung Veterans General Hospital, Taichung, Taiwan; ^4^ Institute of Pharmacology, School of Medicine, National Yang-Ming University, Taipei, Taiwan; ^5^ Agricultural Biotechnology Research Center, Academia Sinica, Taipei, Taiwan; ^6^ Department of Dermatology, Taichung Veterans General Hospital, Taichung, Taiwan; ^7^ Department of Education and Research, Taichung Veterans General Hospital, Taichung, Taiwan; ^8^ Rong Hsing Research Center for Translational Medicine, National Chung Hsing University, Taichung, Taiwan

**Keywords:** imiquimod, aerobic glycolysis, HIF-1α, apoptosis

## Abstract

Tumor cells rely on aerobic glycolysis to maintain unconstrained cell growth and proliferation. Imiquimod (IMQ), a synthetic Toll-like receptor (TLR) 7/8 ligand, exerts anti-tumor effects directly by inducing cell death in cancer cells and/or indirectly by activating cellular immune responses against tumor cells. However, whether IMQ modulates glucose metabolism pathways remains unclear. In this study, we demonstrated that IMQ can enhance aerobic glycolysis by up-regulating HIF-1α expression at the transcriptional and translational levels via ROS mediated STAT3- and Akt-dependent pathways, independent of TLR7/8 signaling. The genetic silencing of HIF-1α not only repressed IMQ-induced aerobic glycolysis but also sensitized cells to IMQ-induced apoptosis due to faster ATP and Mcl-1 depletion. Moreover, the glucose analog 2-DG and the Hsp90 inhibitor 17-AAG, which destabilizes the HIF-1α protein, synergized with IMQ to induce tumor cell apoptosis *in vitro* and significantly inhibited tumor growth *in vivo*. Thus, we hypothesize that the IMQ-induced up-regulation of HIF-1α and aerobic glycolysis is a protective response to the metabolic stress generated by IMQ treatment, and thus, co-treatment with inhibitors of HIF-1α and/or glycolysis may be a useful therapeutic strategy to enhance the anti-tumor effects of IMQ in clinical settings.

## INTRODUCTION

Tumor cells metabolize glucose through different pathways than differentiated and non-transformed normal cells. Tumor cells increase glucose uptake and predominantly utilize glycolysis instead of mitochondrial respiration, even in the presence of abundant oxygen (aerobic glycolysis, the Warburg effect) [1]. This glycolytic switch in tumors provides proliferative benefits because the cells can efficiently use glucose to produce other metabolites for anabolism, and this switch is promoted by reduced oxygen availability (hypoxia) and/or various signaling pathways that are frequently dysregulated during tumor progression [2]. Hypoxia-inducible factor 1 (HIF-1), a heterodimeric transcription factor consisting of the constitutively expressed β subunit (HIF-1β) and the oxygen-regulated α subunit (HIF-1α), is the master regulator of metabolic reprogramming from oxidative respiration to aerobic glycolysis. During hypoxia, HIF-1α is stabilized and protected from proteasomal degradation, allowing it to interact with HIF-1β. The complete HIF-1 protein then translocates to the nucleus to activate downstream gene expression in response to hypoxia [3, 4]. The activation of oncogenic signaling pathways, such as the PI3K/Akt, MAPK/ERK and STAT3 signaling pathways, also promotes HIF-1α expression at the transcriptional and translational levels to increase the rate of glucose utilization in tumors even in the presence of sufficient oxygen [5–7]. In addition, activation of HIF-1α can increase the expression of anti-apoptotic Bcl-2 family members to prevent cell apoptosis [8–10]. Aerobic glycolysis can prevent cells from undergoing apoptosis through the inhibition of mitochondrial respiration, which involves the release of cytochrome c and subsequent caspase cascade activation [11, 12]. Thus, HIF-1α expression and aerobic glycolysis are essential for tumor growth, and the inhibition of both may enhance sensitivity to anti-tumor agents.

Imiquimod (IMQ), a Toll-like receptor (TLR) 7 and 8 ligand, is a synthetic nucleotide-like compound of the imidazoquinoline family that has both anti-tumor and anti-viral activity [13]. IMQ is currently used as a topical, non-invasive treatment for superficial basal cell carcinoma (BCC), viral warts, actinic keratosis, cutaneous squamous cell carcinoma *in situ* and cutaneous metastases of malignant melanoma [14, 15]. IMQ exerts its anti-tumoral activity through the activation of cell-mediated immune responses by stimulating TLR7/8 in dendritic cells and directly by inducing the apoptosis of skin cancer cells in a membrane-death receptor-independent manner [16, 17]. IMQ also induces non-apoptotic, autophagic cell death in Caco-2 colon cancer cells and BCC cell lines [18, 19]. Moreover, IMQ rapidly depletes the Mcl-1 protein in skin cancer cells, and Mcl-1 over-expression may result in resistance to IMQ-induced apoptosis [20]. Thus, these previous studies suggest that IMQ exerts its anti-tumoral activity indirectly by activating immune responses and directly by inducing cell death in tumors. Recently, TLR2, 4 and 9 ligands were reported to modulate glucose metabolism to favor aerobic glycolysis in activated dendritic cells [21]. In addition, the involvement of HIF-1α in TLR7/8-mediated inflammatory response in THP-1 human myeloid macrophage had been reported [22, 23], but whether IMQ can modulate glucose metabolism through HIF-1α in tumor cells remains unclear.

In this study, we demonstrated that IMQ treatment greatly enhanced aerobic glycolysis in tumor cells in a manner independent of TLR7/8 expression. We found that IMQ-induced aerobic glycolysis was regulated by HIF-1α expression. IMQ stimulated STAT3 and PI3K/Akt through ROS to enhance HIF-1α expression at the mRNA and protein levels but did not affect the stability of the HIF-1α protein or its rate of degradation. The genetic silencing of HIF-1α not only reversed IMQ-induced aerobic glycolysis but also sensitized cancer cells to IMQ-induced apoptosis, as a result of rapid ATP depletion and decreased Mcl-1 levels. Finally, the glycolytic inhibitor 2-DG and the Hsp90 inhibitor 17-AAG, which decreases HIF-1α protein stability, synergized with IMQ to induce apoptosis in tumor cells and effectively prevent tumor growth in mouse tumor xenograft models. Our results indicate that IMQ-induced HIF-1α expression and aerobic glycolysis may play protective roles against IMQ-generated metabolic stress, suggesting that co-treatment with inhibitors of HIF-1α or glycolysis and IMQ may provide a novel therapeutic strategy to enhance the anti-tumor effects of IMQ.

## RESULTS

### IMQ enhanced aerobic glycolysis in tumor cells

**Figure 1:**
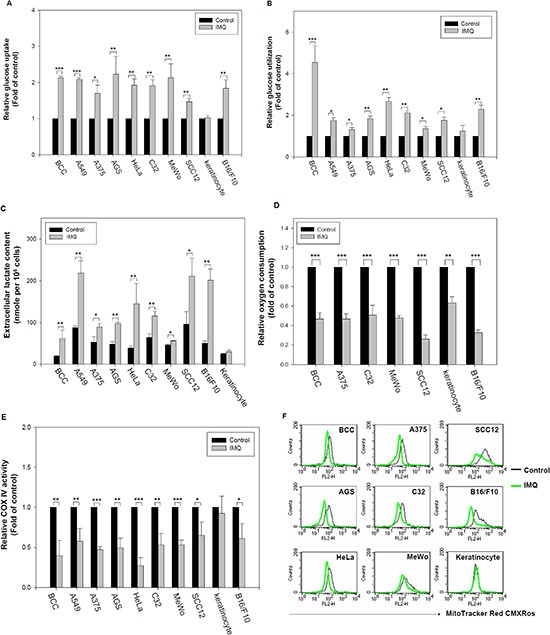
IMQ induced aerobic glycolysis in tumor cells IMQ increased glucose uptake into cells **(A)** and decreased extracellular glucose content **(B)** in tumor cells. BCC, A549, A375, AGS, HeLa, C32, MeWo, SCC12 and B16F10 cells and primary human keratinocytes were incubated in culture medium with or without 50 μg/ml IMQ for 12 hours, and then, the relative glucose uptake and glucose utilization (determined by extracellular glucose content) was analyzed using a glucose uptake assay and glucose content assay with normalization based on the total cell number, respectively. **(C)** IMQ increased lactate secretion by tumor cells. Tumor cell lines were incubated with or without 50 μg/ml IMQ for 24 hours, and then, the lactate content of the culture medium was determined using a lactate content assay with normalization to the total cell number. **(D)** IMQ decreased oxygen consumption by tumor cells. Tumor cell lines were treated with or without 50 μg/ml IMQ, and then, the extracellular oxygen concentration was determined using a polarographic oxygen electrode. **(E)** IMQ reduced mitochondrial respiration in tumor cells. BCC, A549, A375, AGS, HeLa, C32, MeWo, SCC12 and B16F10 cells and primary human keratinocytes were incubated in culture medium with or without 50 μg/ml IMQ for 8 hours, and then harvested cell lysate for cytochrome oxidase activity assay. **(F)** IMQ decreased mitochondrial potential in tumor cells. BCC, AGS, HeLa, A375, MeWo, C32, SCC12, B16F10 and keratinocytes were treated with or without 50 μg/ml IMQ for 8 hours, the cells were assessed by fluorescence due to MitoTracker Red CMXRos staining using flow cytometry. The data are expressed as the mean ± S.E.M. of at least three independent experiments. * *p* < 0.05; ** *p* < 0.01; *** *p* < 0.001.

To explore whether IMQ modulates glucose metabolism in tumor cells, we determined the intracellular glucose uptake, extracellular glucose and lactate contents, which indicate the rate of aerobic glycolysis, before and after IMQ treatment. IMQ significantly increased glucose uptake, glucose utilization and lactate secretion in BCC, A549, AGS, HeLa, SCC12, A375, MeWo, C32 and B16F10 cells but not in primary human keratinocytes (Fig. 1A, 1B and 1C). The switch to aerobic glycolysis from oxidative respiration in cells can be characterized by decreased oxygen consumption and mitochondria respiration. We found that treatment with IMQ reduced the extracellular oxygen consumption and cytochrome oxidase activity in cultures of different cancer cell lines (Fig. 1D and 1E). Consistent with this reduction in mitochondrial respiration, mitochondrial potential also decreased after exposure to IMQ (Fig. 1F). IMQ is a TLR7/8 ligand, and TLR signaling has been reported to modulate glucose metabolism in dendritic cells [21]. To resolve whether the IMQ-induced aerobic glycolysis was mediated by TLR7/8, we examined TLR7 and TLR8 expression in the tumor cell lines and primary human keratinocytes. The expression patterns of TLR7 and TLR8 had no correlation with IMQ-induced aerobic glycolysis in the tested cell lines (Fig. S1A). Thus, we concluded that IMQ-induced aerobic glycolysis is not dependent on TLR7 or TLR8 expression. Taken together, our results indicate that IMQ can enhance aerobic glycolysis in tumor cells and that this process is independent of TLR7 and TLR8 expression.

### IMQ induced HIF-1α expression and activation in tumor cells

**Figure 2:**
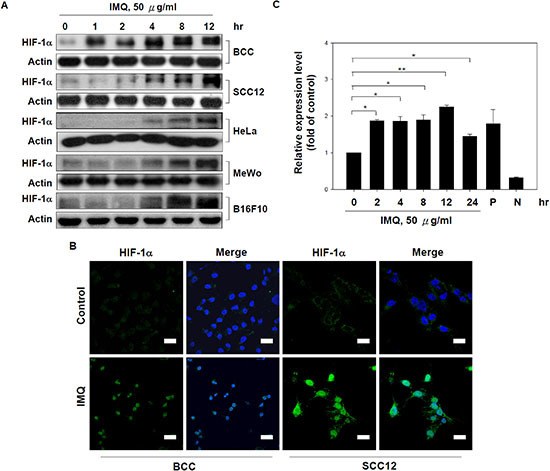
IMQ induced HIF-1α expression and activation in tumor cells **(A)** IMQ increased HIF-1α expression in various tumor cell lines. BCC, SCC12, HeLa, MeWo and B16F10 cells were treated with 50 μg/ml IMQ for 0, 1, 2, 4, 8 or 12 hours and then harvested to prepare cell lysates for immunoblotting with HIF-1α and β-actin antibodies. **(B)** IMQ promoted the nuclear translocation of HIF-1α in tumor cells. BCC and SCC12 cells were treated with or without 50 μg/ml IMQ for 4 hours and then processed for immunocytochemistry using a rabbit anti-HIF-1α antibody and a FITC-conjugated goat anti-rabbit antibody. Scale bars, 20 μm. **(C)** IMQ enhanced HIF-1α transactivation activity. BCC cells were transfected with a HRE-driven luciferase reporter vector for 48 hours and then treated with 50 μg/ml IMQ for 0, 2, 4, 8, 12 or 24 hours. The cell lysates were collected and processed for a luciferase assay by the detection of luminescence. The P and N groups were the cells transfected with the positive control vector and the negative control vector, respectively. The data are expressed as the mean ± S.E.M. of at least three independent experiments. * *p* < 0.05; ** *p* < 0.01.

HIF-1α has been demonstrated to be the master regulator of the switch in glucose metabolism from oxidative respiration to aerobic glycolysis [3, 4]. Resiquimod and ssRNA, another TLR7/8 ligands, induced HIF-1α expression in THP-1 cells [23]. We hypothesized that IMQ induces HIF-1α expression to shift glucose metabolism to aerobic glycolysis in normoxic condition. As shown in Fig. 2A and Fig. S2A, IMQ increased HIF-1α protein expression in BCC, SCC12, HeLa, MeWo and B16F10 cells in a time- and dose-dependent manner. Previously studies have shown that activated HIF-1α translocates to the nucleus and activates the expression of its target genes, such as GLUT1 and VEGF [24, 25]. Thus, it was important to determine whether IMQ stimulated HIF-1α activation in tumor cells. We observed that IMQ not only promoted the nuclear translocation of HIF-1α (Fig. 2B) but also increased the mRNA levels of VEGF and GLUT1 in BCC cells (Fig. S2B). We also confirmed that IMQ enhanced HRE-driven luciferase expression in BCC cells in time course experiments (Fig. 2C). These results indicate that IMQ stimulated HIF-1α expression and activated the expression of its target genes in tumor cells.

### IMQ induced HIF-1α expression via transcriptional and translational control in tumor cells

**Figure 3:**
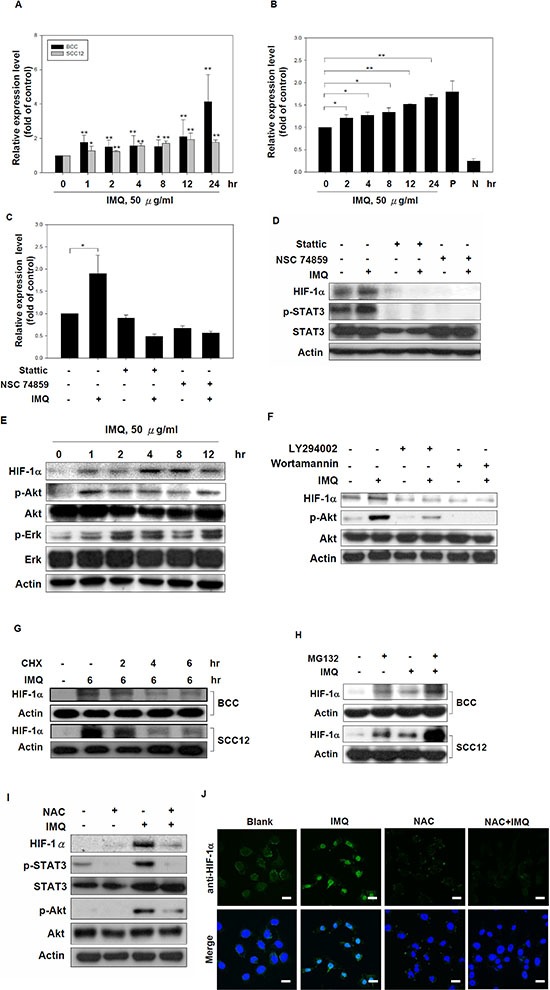
IMQ increased HIF-1α expression at the transcriptional and translational levels **(A)** IMQ stimulated HIF-1α mRNA expression in tumor cells. BCC and SCC12 cells were treated with 50 μg/ml IMQ for 0, 1, 2, 4, 8, 12 or 24 hours, and then, the HIF-1α mRNA levels were determined by quantitative real-time PCR. **(B)** IMQ enhanced STAT3 transcriptional activity in BCC cells. BCC cells were transfected with a STAT3-driven luciferase reporter vector for 48 hours. The cells were treated with 50 μg/ml IMQ for 0, 2, 4, 8, 12 or 24 hours and then analyzed by a luciferase assay. **(C** and **D)** The inhibition of STAT3 reduced the HIF-1α mRNA **(C)** and protein levels **(D)** in IMQ-treated cells. BCC cells were pre-treated with Stattic (10 μM) or NSC74859 (1 μM) for 1 hour and then treated with or without 50 μg/ml IMQ for 4 hours. Cell lysates were collected, and the mRNA and protein levels were determined by quantitative real-time PCR and immunoblotting with HIF-1α, p-STAT3, STAT3 and β-actin antibodies, respectively. **(E)** IMQ-induced HIF-1α expression was associated with the activation of the Akt and ERK signaling pathways. BCC cells were incubated with medium containing 50 μg/ml IMQ for 0, 1, 2, 4, 8 or 12 hours, and cell lysates were then harvested for immunoblotting with HIF-1α, phosphorylated-Akt, Akt, phosphorylated-ERK, ERK and β-actin antibodies. **(F)** Suppression of the PI3K/Akt signaling pathway reduced IMQ-induced HIF-1α expression. BCC cells were incubated for 1 hour with 20 μM LY294002 or 10 μM wortmannin in medium and then treated with or without 50 μg/ml IMQ for 4 hours. Cell lysates were collected for immunoblotting with HIF-1α, p-Akt, Akt and β-actin antibodies. **(G)** IMQ treatment did not increase the protein stability of HIF-1α. BCC and SCC12 cells were treated with IMQ (50 μg/ml) for 6 hours with or without co-treated with CHX (10 μg/ml) for 0, 2, 4 or 6 hours. Cell lysates were prepared for immunoblotting with HIF-1α and β-actin antibodies. **(H)** IMQ did not affect the proteasomal degradation of the HIF-1α protein. BCC and SCC12 cells were pre-treated with MG132 (10 μM) for 1 hour, treated with IMQ (50 μg/ml) for 4 hours and then processed for immunoblotting with HIF-1α and β-actin antibodies. **(I)** The depletion of ROS resulted in the down-regulation of HIF-1α expression with the repression of the STAT3 and PI3K/Akt signaling pathway. BCC cells were incubated with medium containing 2 mM NAC for 30 minutes and then treated with 50 μg/ml IMQ for 4 hours. Cell lysates were collected for immunoblotting with HIF-1α, p-Akt, Akt, p-STAT3, STAT3 and β-actin antibodies. **(J)** The inhibition of ROS production repressed IMQ-induced HIF-1α nuclear translocation. BCC cells were pre-treated with 2 mM NAC for 30 minutes and then treated with 50 μg/ml IMQ for 4 hours. The subcellular localization of HIF-1α was determined by immunocytochemistry. Scale bars, 20 μm. The data are expressed as the mean ± S.E.M. of at least three independent experiments. * *p* < 0.05; ** *p* < 0.01.

Next, we evaluated the control mechanisms of IMQ-induced HIF-1α expression in tumor cells. As shown in Fig. 3A, we found that IMQ induced HIF-1α mRNA expression in a time-dependent manner in BCC and SCC12 cells. IMQ has been reported to induce STAT3 phosphorylation [26], and activated STAT3 can increase HIF-1α expression through its transcriptional activation [27]. We examined whether IMQ induces STAT3 transcriptional activation using a STAT3 reporter plasmid and whether IMQ-induced HIF-1α transcription is mediated by STAT3 signaling using the STAT3 inhibitors Stattic and NSC74859. First, IMQ induced STAT3-driven luciferase expression in BCC cells in a time-dependent manner (Fig. 3B). Second, the STAT3 inhibitors not only significantly inhibited IMQ-induced HIF-1α mRNA expression but also decreased the HIF-1α protein level in BCC and SCC12 cells (Fig. 3C, Fig. 3D and Fig. S3A). Activation of the MAPK/ERK and Akt/mTOR pathways has been reported to enhance HIF-1α protein synthesis [6, 7]. We also observed that IMQ treatment promoted the phosphorylation of ERK1/2 and Akt in BCC and SCC12 cells, indicating that the MAPK/ERK and Akt/mTOR signaling pathways were activated (Fig. 3E and Fig. S3B). The PI3K inhibitors LY294002 and wortmannin efficiently inhibited IMQ-induced HIF-1α expression (Fig. 3F), but the inhibition of ERK by PD98059 or U0126 had no effect on IMQ-induced HIF-1α expression in BCC cells (Fig. S3C). To determine whether IMQ affects HIF-1α protein stability in tumor cells, protein synthesis was inhibited with CHX. CHX treatment decreased HIF-1α protein levels in a time-dependent manner in IMQ-treated BCC and SCC12 cells (Fig. 3G). Furthermore, HIF-1α has been reported to be degraded via the ubiquitin-proteasome pathway [28]. We showed that treatment with the proteasome inhibitor MG132 resulted in significantly higher HIF-1α protein levels in IMQ-treated BCC and SCC12 cells compared with IMQ or MG132 treatment alone, indicating that HIF-1α was still degraded by the proteasome during IMQ stimulation (Fig. 3H). Thus, we concluded that the IMQ-induced increase in the HIF-1α protein level is primarily regulated at the transcriptional and translational levels and not at the level of protein stability or proteasomal degradation. We also observed that IMQ increased cellular ROS production (Fig. S3D), and various studies have reported that ROS can stimulate STAT3 and Akt activation in cells [29–32]. To test whether IMQ activates STAT3 and Akt via ROS production, we pre-treated the cells with the potent ROS scavenger N-acetylcysteine (NAC) before IMQ treatment. We observed that treatment with NAC significantly reduced the levels of HIF-1α, phosphorylated STAT3 and phosphorylated Akt in both BCC and SCC12 cells (Fig. 3I and Fig. S3E). In addition, treatment with NAC decreased the nuclear translocation of HIF-1α in IMQ-treated BCC and SCC12 cells (Fig. 3J and Fig. S3F). Taken together, these results indicate that IMQ may activate STAT3 to increase HIF-1α mRNA expression and may stimulate Akt to promote HIF-1α protein synthesis through ROS in tumor cells.

### The induction of HIF-1α mediated IMQ-induced aerobic glycolysis in tumor cells

**Figure 4:**
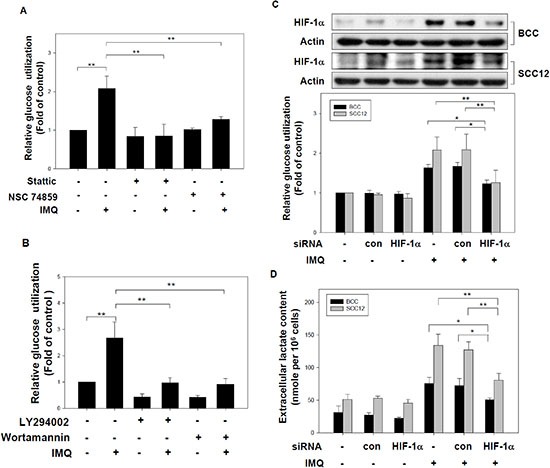
The induction of HIF-1α mediated IMQ-induced aerobic glycolysis in tumor cells **(A)** The disruption of STAT3's function inhibited IMQ-induced glucose utilization. BCC cells were incubated in medium containing 10 μM Stattic or 1 μM NSC74859 for 1 hour and then treated with or without 50 μg/ml IMQ for 12 hours. The glucose content assay was performed to determine the relative extracellular glucose content normalized to the total cell number. **(B)** Inhibition of the PI3K/Akt signaling pathway repressed IMQ-induced glucose utilization. BCC cells were pre-treated with 20 μM LY294002 or 10 μM wortmannin and then treated with or without 50 μg/ml IMQ. The relative glucose utilization level was calculated using a glucose content assay with normalization to the total cell number. (C) and (D) The genetic silencing of HIF-1α suppressed IMQ-induced glucose utilization and lactate production in tumor cells. BCC and SCC12 cells were transfected with a HIF-1α-specific siRNA or a non-specific control siRNA for 24 hours. The transfected cells were treated with 50 μg/ml IMQ. The HIF-1α protein was checked by immunoblotting (up panel of figure C) and the relative glucose utilization level over 12 hours was determined using a glucose content assay (low panel of figure C). The extracellular lactate levels of transfected cells treated with 50 μg/ml IMQ for 24 hours were analyzed using a lactate content assay **(D)**. These results were normalized to the total cell number. The data are expressed as the mean ± S.E.M. of at least three independent experiments. * *p* < 0.05; ** *p* < 0.01.

We observed IMQ-induced aerobic glycolysis and HIF-1α expression in several tumor cell lines. Thus, it is necessary to investigate the relationship between aerobic glycolysis and HIF-1α expression in IMQ-treated cells. We showed that the inhibition of STAT3 by Stattic and NSC74859 not only reduced IMQ-induced HIF-1α expression but also significantly repressed glucose utilization in IMQ-treated BCC cells (Fig. 4A). In addition, treatment with LY294002 and wortmannin significantly reduced glucose utilization in IMQ-treated BCC cells (Fig. 4B). However, PD98059 and U0126 had no influence on glucose utilization by IMQ-treated BCC cells (Fig. S4). These parallel changes indicate that the inhibitors that suppressed IMQ-induced HIF-1α expression also inhibited IMQ-enhanced aerobic glycolysis. Furthermore, the specific knock-down of HIF-1α expression by HIF-1α siRNA significantly decreased glucose utilization and lactate secretion in IMQ-treated BCC and SCC12 cells (Fig. 4C and Fig. 4D). These data provide evidence that IMQ-induced aerobic glycolysis is mediated by HIF-1α expression in tumor cells.

### The induction of HIF-1α mediated IMQ-induced apoptosis in tumor cells

**Figure 5:**
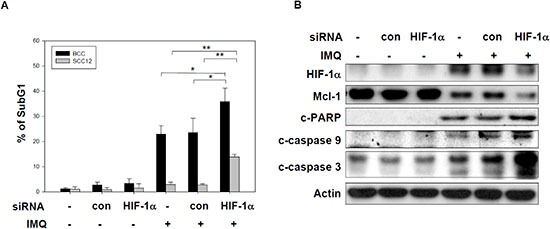
HIF-1α expression are involved in IMQ-induced apoptosis in tumor cells **(A** and **B)** The knock-down of HIF-1α sensitized tumor cells to IMQ-induced apoptosis. **(A)** BCC and SCC12 cells were transfected with a HIF-1α specific siRNA or a non-specific siRNA for 24 hours and then treated with 50 μg/ml IMQ for 24 or 48 hours, respectively. The cells were then analyzed with a DNA content assay based on PI staining and flow cytometry. **(B)** The siRNA transfected BCC cells incubated with 50 μg/ml IMQ for 12 hours were analyzed by immunoblotting with cleaved-PARP, cleaved-caspase 9, cleaved-caspase 3, Mcl-1 and β-actin antibodies. The data are expressed as the mean ± S.E.M. of at least three independent experiments. * *p* < 0.05; ** *p* < 0.01.

It has been reported that the induction of HIF-1α promotes glycolysis and Mcl-1 expression via its transcriptional activity to protect against cell apoptosis [10]. Our previous studies demonstrated that IMQ reduced Mcl-1 protein expression to induce intrinsic apoptosis in skin cancer cells [20]. To determine the relationship between the induction of HIF-1α and apoptosis in IMQ-treated tumor cells, we used HIF-1α siRNA to reduce HIF-1α expression and then determined the level of apoptosis in IMQ-treated cells. HIF-1α knock-down increased the apoptotic cell population relative to that in the control siRNA groups for IMQ-treated BCC and SCC12 cells, although SCC12 cells were relative resistant to IMQ-induced apoptosis (Fig. 5A). HIF-1α knock-down also enhanced the levels of apoptotic markers such as cleaved-PARP, cleaved-caspase 3 and cleaved-caspase 9 and reduced the Mcl-1 protein level in IMQ-treated BCC and SCC12 cells (Fig. 5B and Fig. S5). Our results suggest that the inhibition of HIF-1α expression may sensitize the IMQ-induced apoptosis in cancer cells.

### The inhibition of aerobic glycolysis and HIF-1α expression synergistically increased IMQ-induced apoptosis *in vitro* and *in vivo*

**Figure 6:**
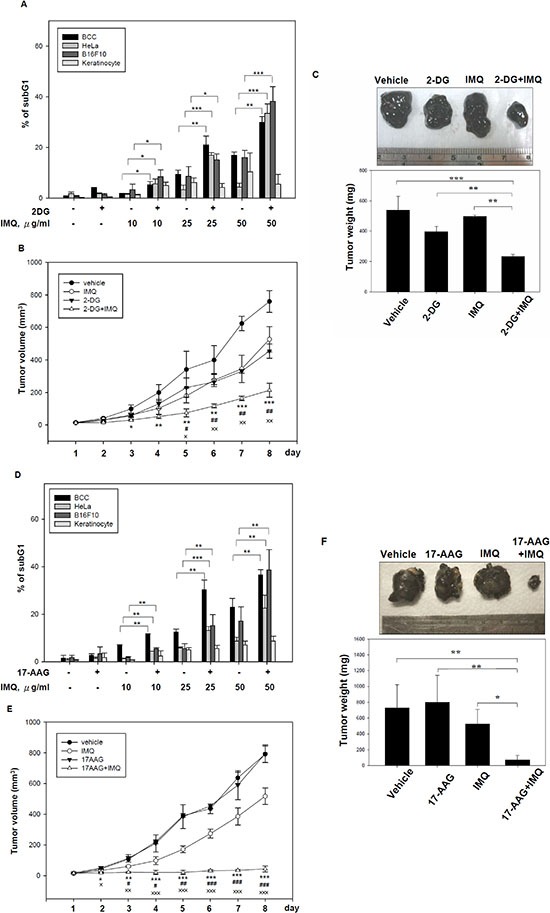
The combination of a pharmacological glycolytic inhibitor 2-DG or a Hsp90 inhibitor 17-AAG with IMQ significantly suppressed tumor growth in *in vitro* and *in vivo* **(A)** The glucose analog 2-DG enhanced IMQ-induced apoptosis in tumor cells but not in normal cells. BCC, HeLa and B16F10 cells and primary human keratinocytes were treated with sub-lethal dose of 2-DG and co-treated with 10, 25 or 50 μg/ml IMQ for 24 hours. The cells were collected, and the hypodiploid cell population was detected using a DNA content assay based on flow cytometry. The sub-lethal dose of 2-DG in BCC cells and primary human keratinocytes was 50 mM, and the sub-lethal dose in HeLa and B16F10 cells was 5 mM. **(B** and **C)** 2-DG synergized with IMQ to repress melanoma growth in a mouse tumor xenograft model. C57BL/6 mice were subcutaneously injected with 2 × 10^5^ B16F10 mouse melanoma cells. After the tumor area reached 0.2 mm^2^, 2-DG (62.5 mg/kg), IMQ (25 mg/kg) or both were injected daily into the tumor site, and the tumor volume was measured for one week **(B)**. After 8 days of treatment, the animals were sacrificed, and the tumor weights were recorded **(C)**. **(D)** The pharmacological inhibition of HIF-1α enhanced IMQ-induced apoptosis in tumor cells. BCC, HeLa and B16F10 cells and primary human keratinocytes were pre-treated with 10 μM 17-AAG for 1 hour and then incubated with culture medium containing IMQ (0, 10, 25, 50 μg/ml) for 24 hours. The cells were then collected for use in a DNA content assay based on flow cytometry. **(E** and **F)** The 17-AAG synergized with IMQ to suppress tumor growth *in vivo*. B16F10 melanoma-bearing mice were generated as previously described and treated daily with 40 mg/kg 17-AAG, 25 mg/kg IMQ or a combination of 17-AAG and IMQ by *in situ* injection at the tumor site. The tumor volume was measured daily for one week **(E)**. All the animals were sacrificed after 8 days of treatment, and the tumor weights were recorded **(F)**. The asterisk represents significance between control and 2-DG + IMQ (B), control and 17-AAG+IMQ (E). The pound represents significance between IMQ and 2-DG + IMQ (B), IMQ and 17-AAG+IMQ (E); the double dagger represents significance between 2DG and 2DG+IMQ (B), 17-AAG and 17-AAG+IMQ (E). The data are expressed as the mean ± S.E.M. of at least three independent experiments (* *p* < 0.05; ** *p* < 0.01; *** *p* < 0.001; # *p* < 0.05; ## *p* < 0.01; ### *p* < 0.001; × *p* < 0.05; ×× *p* < 0.01).

The inhibition of aerobic glycolysis or HIF-1α expression using genetic manipulation or chemical inhibitors may sensitize tumor cells to chemotherapy [33–36]. Next, we used the nonmetabolizable glucose analog 2-DG and the Hsp90 inhibitor 17-AAG, a derivative of geldanamycin (GA) which had been reported to not only destabilizes the HIF-1α protein but also disrupts STAT3- HIF-1α activation axis [37, 38], along with IMQ co-treatment in cancer cells and xenograft tumor mouse models to test this hypothesis. As shown in Fig. 6A and S6A, 2-DG synergistically enhanced IMQ-induced apoptosis and decreased cell viability in BCC, HeLa and B16F10 cells but not in primary human keratinocytes. We also observed that the combination 2-DG and IMQ significantly suppressed tumor growth relative to the level of suppression achieved by treatment with a single agent in B16F10 tumor-bearing mice (Fig. 6B and Fig. 6C). In addition, 17-AAG co-administered with IMQ significantly increased the apoptotic cell content and reduced cell viability in BCC, HeLa and B16F10 cells but had no effect on primary human keratinocytes (Fig. 6D and Fig. S6B). Moreover, treatment with 17-AAG effectively blunted the IMQ induced STAT3 phosphorylation and HIF-1α protein expression (Fig. S6C). Consistently, we also found that IMQ dramatically repressed tumor growth when co-administered with 17-AAG to B16F10 tumor-bearing mice (Fig. 6E and Fig. 6F). Taken together, these results indicate that the disruption of glucose utilization by 2-DG and the targeting of HIF-1α protein by 17-AAG can enhance the antitumor activity of IMQ both *in vitro* and *in vivo*.

## DISCUSSION

No previous studies have shown that the TLR7 agonist IMQ modulates glucose metabolism in tumor cells. In this study, we demonstrated that IMQ increased glucose uptake, glucose utilization and lactate secretion and reduced mitochondria respiration, indicating that IMQ increased the rate of aerobic glycolysis, in both TLR7/8-expressing and TLR7/8-non-expressing tumor cells. We found that IMQ induced HIF-1α expression and nuclear translocation and activated its downstream genes, including VEGF and GLUT1. Pharmacological inhibition of the PI3K/Akt or STAT3 pathway significantly reduced IMQ-induced glucose utilization and IMQ-induced HIF-1α expression at the transcriptional and translational levels. The genetic silencing of HIF-1α expression reduced the IMQ-enhanced glucose utilization and sensitized tumor cells to IMQ-induced apoptosis. Furthermore, IMQ synergized with the glucose analog 2-DG and the 17-AAG which destabilizes the HIF-1α protein to induce apoptosis in tumor cells but not in primary human keratinocytes. The combination of IMQ with 2-DG or 17-AAG also significantly suppressed melanoma xenograft growth relative to the levels of growth observed for treatment with a single agent *in vivo*.

A recent study demonstrated that TLR2, 4 and 9 agonists stimulated the PI3K/Akt pathway to shift glucose metabolism to aerobic glycolysis, which was essential for the survival and activation of dendritic cells [21]. In the present study, we demonstrated that the TLR7 ligand IMQ increased glycolysis and decreased mitochondria respiration in both TLR7/8-positive and TLR7/8-negative cell lines. These results indicate that IMQ-enhanced aerobic glycolysis may not depend on TLR7/8 expression in tumor cells. Interestingly, it has been shown that IMQ can interact with the adenosine receptor to induce proinflammatory cytokines in TLR7/8-negative cells [39]. A derivative of IMQ lacking TLR7/8 activity has also been shown to stimulate cytokine production [40]. Thus, it is possible that some IMQ-induced responses may not require the presence of TLR7/8. Although different TLR7/8 ligands, resiquimod and ssRNA, have been reported to up-regulate HIF-1α expression and induce TLR7/8-mediated inflammatory response in TLR7/8 expressing-THP1 cells [22, 23], we observed that over-expression of TLR7 but not TLR8 could further enhance the IMQ-enhanced aerobic glycolysis in BCC cells (Fig. S1B, S1C and S1D). Thus, IMQ-enhanced aerobic glycolysis may occur in TLR7/8 non-expressed tumor cells, however, the expression of TLR7 may strengthen this effect. The other receptors that modulate IMQ-enhanced aerobic glycolysis should be further evaluated.

Under hypoxic conditions, stabilized HIF-1α dimerizes with HIF-1α, translocates to the nucleus and transactivates the expression of its downstream genes, which encode glucose transporters and all glycolytic enzymes, to switch the metabolic pathway to aerobic glycolysis for energy production. HIF-1 also up-regulates VEGF expression to promote angiogenesis [25, 41]. In this study, we demonstrated that IMQ induced HIF-1α protein expression and nuclear translocation, increased HRE-driven luciferase reporter activity and enhanced the expression of genes downstream of HIF-1, including VEGF and GLUT1, under normoxic conditions. This IMQ-induced HIF-1α expression also occurred in TLR7/8-negative tumor cells, although it has been reported previously that the ligand-induced activation of TLR7/8 leads to the accumulation of HIF-1α protein in THP-1 human myeloid macrophages [22, 23]. The level of HIF-1α protein can be regulated at several levels, including transcription, translation and degradation [42]. STAT3 and NF-κB increase HIF-1α expression via their transcriptional activity [27, 43, 44], activation of the PI3K/Akt and MAPK/ERK pathways promotes HIF-1α protein synthesis [6, 7], and hypoxia stabilizes the HIF-1α protein by protecting it from proteasomal degradation [28]. In our study, we observed STAT3 inhibitors, but not an NF-κB inhibitor (Fig. S7A), reduced both the HIF-1α mRNA and protein levels in IMQ-treated cells. In THP-1 cells, the PI3K/Akt and MAPK/ERK pathways have been reported to not be involved in TLR7/8-mediated HIF-1α accumulation [23]. In contrast, we observed that the inhibition of the PI3K pathway, but not ERK signaling, not only inhibited IMQ-enhanced aerobic glycolysis but also dramatically suppressed IMQ-induced HIF-1α expression in TLR7/8-negative BCC cells. Thus, we hypothesized that IMQ induces HIF-1α expression by increasing transcription and promoting translation through activation of the STAT3 and PI3K/Akt pathways, respectively. Previous studies have demonstrated that ROS can inhibit PHD1-3 to prevent HIF-1α degradation via the ubiquitin-proteasome pathway under normoxic conditions [45]. However, ROS also stimulate the PI3K/Akt pathway and induce STAT3 phosphorylation in cells [29–32]. We found that IMQ induced ROS production (Fig. S3D). Treatment of IMQ-treated cells with the ROS scavenger NAC decreased the levels of phosphorylated-STAT3 and phosphorylated-Akt in addition to down-regulating HIF-1α. Moreover, in IMQ-treated BCC cells, we found that CHX treatment decreased the HIF-1α protein level and that MG132 treatment resulted in a much higher level of HIF-1α protein. Thus, we hypothesized that IMQ-induced ROS activate both the STAT3 and PI3K/Akt pathways to promote HIF-1α expression but do not increase the stability or inhibit the proteasomal degradation of HIF-1α in tumor cells. Based on our results, we concluded that IMQ activates the STAT3 and PI3K/Akt pathways to increase HIF-1α expression at both the transcriptional and translational levels in TLR7/8-negative cancer cells. IMQ-induced ROS generation may play an important role in this process. Importantly, the signaling pathways involved in IMQ-induced HIF-1α accumulation may differ between TLR7/8-positive cells and TLR7/8-negative cells.

Aerobic glycolysis has been reported to be induced by PI3K/Akt and MAPK/ERK signaling [46, 47]. The results for the pharmacological inhibition of the Akt and ERK pathways suggest that IMQ activates PI3K/Akt to enhance aerobic glycolysis but does not affect MAPK/ERK signaling. IMQ is known to activate STAT3 [26], and STAT3 has also been reported to act as a master regulator of the induction of aerobic glycolysis [5, 26, 27]. We found that inhibition of IMQ-stimulated phosphorylated-STAT3 using STAT3 inhibitors resulted in a reduced rate of glucose utilization. Similar to the signaling for IMQ-induced HIF-1α accumulation, we demonstrated that IMQ activated the PI3K/Akt and STAT3 pathways to enhance aerobic glycolysis in TLR7/8-negative tumor cells. Also demonstrating that aerobic glycolysis is controlled by HIF-1α in IMQ-treated cancer cells, the genetic silencing of HIF-1α reversed the IMQ-enhanced glucose utilization and lactate production. HIF-1α knock-down also sensitized tumor cells to IMQ-induced apoptosis, with the rapid depletion of the Mcl-1 protein. The induction of HIF-1α upregulates Mcl-1 expression via its transcriptional activity [10]. This data corresponds to our previous finding that Mcl-1 mRNA levels were upregulated during IMQ treatment. However, the IMQ induced decrease in Mcl-1 protein level is not due to transcriptional or degradation control but is mainly mediated by translation inhibition [20]. Thus, as compared with control siRNA knock-down, it is reasonable to observe that HIF-1α knock-down inhibited Mcl-1 mRNA transcription and then strongly and rapidly disrupted the Mcl-1 protein translation in BCC and SCC12 cells with IMQ treatment. Interestingly, IMQ dramatically depleted the intracellular ATP content in the BCC, A375, HeLa and A549 cell lines, and the knock-down of HIF-1α resulted in much lower ATP levels in IMQ-treated BCC cells (Fig. S7B and Fig. S7C). Recent studies have demonstrated that the presence of abundant glucose for glycolysis can maintain Mcl-1 expression to protect cells from apoptosis [48]. These results may indicate that the induction of HIF-1α in IMQ-treated cancer cells may promote survival by uptaking more glucose and maintaining a higher intracellular ATP level to protect against apoptosis via switching to aerobic glycolysis. The depletion of ATP results in the activation of the intracellular energy sensor AMP-activated protein kinase (AMPK), which can promote glucose utilization, glycolysis and oxidative respiration to generate more ATP [49]. It is unlike that IMQ enhanced aerobic glycolysis is mediated by AMPK, because of increasing lactate secretion and inhibiting oxygen consumption in IMQ treated cancer cells. Whether IMQ induced AMPK activation and the effect of AMPK activation on glucose metabolism in cancer cells need further investigation. Along with HIF-1α, both hexokinase II and PKM2 have been shown to induce aerobic glycolysis in cells [50, 51]. As shown in Fig. S7D, we found that IMQ did not modulate the hexokinase II or PKM2 expression levels in BCC cells. Thus, we excluded the possibility that hexokinase II and PKM2 are involved in IMQ-induced aerobic glycolysis. Taken together, we hypothesized that induction of HIF-1α promotes the switch to aerobic glycolysis to maintain the intracellular ATP level, thereby gaining the survival advantage in IMQ-treated cancer cells.

Tumors rely on aerobic glycolysis and therefore require more glucose than normal differentiated cells to provide growth and survive advantages for tumor progression [1, 52]. This metabolic switch could be regulated by the activation of HIF-1α during tumor progression [2, 53]. Recently, chemotherapeutic drug, doxorubicin, has been reported to induces HIF-1α accumulation, enhanced VEGF secretion and stimulated tumor angiogenesis in normoxic condition [54]. This study suggested that HIF-1α is a major determinant for cancer cell homeostasis under cytotoxic stress, and targeting aerobic glycolysis and HIF-1α may be a useful and specific strategy for chemotherapy [36]. In the present study, we used the glucose analog 2-DG and the pharmacological Hsp90 inhibitor 17-AAG, which destabilizes the HIF-1α protein and disrupts STAT3-HIF-1α activation axis, to limit IMQ-induced aerobic glycolysis to enhance the anti-tumor activity of IMQ. We found that both 2-DG and 17-AAG synergized with IMQ to induce cell apoptosis in various tumor cell lines but not in normal primary human keratinocytes. We concluded that the combination of IMQ with 2-DG or 17-AAG is a promising strategy for the efficient elimination of tumor cells. In melanoma xenograft-bearing mice, we found that 2-DG and 17-AAG significantly reduced tumor sizes and weights when co-administered with IMQ relative to those in animals treated with a single agent. In addition, the dose of 2-DG used (62.5 mg/kg) has been reported to be a low dose *in vivo* and in clinical trials [55, 56], suggesting that the use of this dose should be safe for tumor therapy in combination with IMQ *in vivo*. However, in our study, the administration of 17-AAG (40 mg/kg/daily per week) resulted in approximately 22% lethal toxicity *in vivo* even when co-administered with IMQ. A recent report showed that 17-AAG at 9 mg/kg/week exerted 10% lethal toxicity in mice, and the recommended safe dosage of 17-AAG in phase I clinical trials is 40 mg/kg/week [57]. Therefore, it is necessary to determine the safe dosage of 17-AAG when used in combination with IMQ for tumor therapy in the future.

Hsp90 inhibitor 17-AAG exert their effects by specifically interacting with the N-terminal ATP binding domain and inhibiting the intrinsic ATPase activity of Hsp90 which is critical for its chaperone function [58]. By inactivating Hsp90, 17-AAG destabilizes Hsp90 client proteins including a wide variety of oncogenic kinases and signaling intermediates. Thus, it is possible that, in addition to destabilize HIF-1α, 17-AAG may simultaneously destabilize other oncoproteins and enhances the antitumor activity of IMQ. However, we suggest the inhibition of HIF-1α expression and/or destabilization of HIF-1α using genetic manipulation or chemical inhibitors may sensitize tumor cells to IMQ-induced apoptosis for the following reasons. First, we demonstrated that the inhibition of HIF-1α expression by RNAi strategy may sensitize the IMQ-induced apoptosis in cancer cells. Second, we demonstrated that 17-AAG effectively blunted the IMQ induced STAT3 phosphorylation and HIF-1α protein accumulation in cancer cell lines. Consistent with our results, it has been found that the 17-AAG not only destabilizes the HIF-1α protein in prostate cancer cells but also disrupts STAT3- HIF-1α activation axis in pancreatic and gastric cancer cells [37, 38]. Third, we provided evidences that chetomin, which inhibits the HIF-1 transactivating activity by disrupting the interactions of the C-terminal transactivation domain of HIF-1α with the CH1 domain of transcriptional coactivator p300 [59, 60], can also enhance the antitumor activity of IMQ both *in vitro* and *in vivo* (Fig. S8). Collectively, different lines of evidence argue that targeting of HIF-1α may disrupt IMQ-induced HIF-1α up-regulation and activity, counteract the IMQ-enhanced aerobic glycolysis and enhance the IMQ-induced apoptosis in cancer cells. 17-AAG can selectively sensitize cancer cells to chemotherapy. However, accumulated evidences indicated that GA and 17-AAG also protect certain cells against specific chemotherapy [58, 61, 62]. Dual effects of GA and 17-AAG had been mention that these agents have cytoprotective response for chemotherapy in apoptosis-prone cells. In contrast, they could sensitize apoptosis-reluctant cancer cells that are dependent on Hsp90 client oncogenic kinases and antiapoptotic proteins [62]. Interestingly, bimodal effects of 17-AAG in the same cancer cells had been observed that low-dose treatment (5-30 nM) with 17-AAG increased and high-dose treatment (1-3 μM) reduced hypoxic HIF-1α protein and activity, respectively [63]. Thus, these results suggest that dosage and cell type specificity will be critical factors in the treatment of tumor with 17-AAG and IMQ combination.

In this study, we demonstrated that the TLR7 ligand IMQ induces aerobic glycolysis in tumor cells in a manner independent of TLR7/8 expression. Second, IMQ induced ROS production, thereby increasing HIF-1α expression at the transcriptional and translational levels via the PI3K/Akt and STAT3 pathways, which triggered aerobic glycolysis in tumor cells. Third, the induction of HIF-1α mediated IMQ-induced apoptosis in tumor cells. Finally, we demonstrated that the inhibition of both glycolysis and HIF-1α expression is a useful strategy to enhance the anti-tumor activity of IMQ *in vitro* and *in vivo*. We believe that this strategy may be applied in clinical trials in the future.

## MATERIALS AND METHODS

### Materials

Propidium iodide (PI) was obtained from Molecular Probes (Carlsbad, CA, USA). TRIzol reagent, LY294002, wortmannin, PD98059, U0126, Stattic, NSC74859, PDTC, cyclohexamide (CHX), MG132, 2-DG, 17-AAG and chetomin were obtained from Sigma (St. Louis, MO, USA). Imiquimod was obtained from InvivoGen (San Diego, CA, USA). Antibodies specific to phospho-STAT3, STAT3, cleaved-caspase 3, cleaved-caspase 9, cleaved-PARP, HIF-1α, phospho-Ser473 Akt, Akt, phospho-ERK1/2 (Thr202/Tyr204) and ERK1/2 were purchased from Cell Signaling Technology (Danvers, MA, USA). Antibodies specific to Mcl-1 and β-actin were purchased from Santa Cruz (Santa Cruz, CA, USA).

### Cell culture

The human melanoma cell lines MeWo, C32 and A375 were cultured in MEM medium supplemented with 10% FBS. The human basal cell carcinoma cell line BCC/KMC-1, the human lung adenocarcinoma cell line A549 and the human gastric cancer cell line AGS were cultured in RPMI medium supplemented with 10% FBS. The human squamous cell carcinoma cell line SCC12 was cultured in F12/DMEM supplemented with 10% FBS. The human cervical carcinoma cell line HeLa and the mouse melanoma cell line B16F10 were cultured in DMEM supplemented with 10% FBS. Primary human keratinocytes from freshly excised neonatal foreskins were purchased from GIBCO (Carlsbad, CA, USA). The keratinocytes were maintained in flasks coated with type IV collagen (Sigma) and cultured in serum-free keratinocyte growth medium (Clonetics, San Diego, CA, USA).

### Extracellular glucose and lactate content assay

The effects of glucose utilization and lactate production in IMQ-treated and untreated cells were analyzed using a Glucose Assay Kit and a Lactate Assay Kit (Sigma) according to the manufacturer's protocols, and the results were normalized to the cell number. In brief, the cells were maintained in 6-well plates and treated with or without IMQ. After the indicated time points, 50 μl of medium was collected and mixed with 50 μl of the prepared glucose or lactate assay mixture (from the Glucose Assay Kit or Lactate Assay Kit, respectively). These samples were then incubated at 37°C in the dark for 30 minutes. The results were quantified using a glucose standard (from the glucose assay kit). The absorbance of each sample was measured using an ELISA plate reader at 570 nm, and the glucose content normalized to the total cell number for the untreated controls was used as the baseline for calculating the fold change. The fold changes in the glucose level were then calculated for the treated groups. The lactate content was normalized to the total cell number for 10^6^ cells, and the data are presented as nmol/10^6^ cells.

### Glucose uptake assays

The effect of glucose uptake in IMQ-treated and untreated cells were analyzed by Glucose Uptake Colorimetric Assay Kit (BioVision, Milpitas, CA, USA.) that was performed according to manufacturer's instruction and normalized with cell number. In brief, the cells were maintained in 96-well plates with 1 mM 2-DG and treated with or without IMQ. After the indicated time points, the cells were lysed by Extraction Buffer, freeze and thaw once then incubated at 85°C for 40 minutes, cool the cell lysate and then adding Neutralization Buffer, spin and diluted the samples 1:10 times by adding Assay Buffer. The diluted samples were mixed with Enzyme Mix, incubated at 37°C for 1 hour, and then stopped the reaction by heating at 85°C for 40 minutes. The samples were added with Glutathione Reductase and DTNB substrate mixture for 5 minutes, and then read the absorbance of OD 512 nm. The intracellular 2-DG content of the untreated controls which normalized to the total cell numbers was used as the baseline for calculating the fold change.

### Oxygen consumption assay

The oxygen consumption of whole cells was measured using a polarographic oxygen electrode (MT200/MT200A Respirometer Cell, StrathKelvin Instruments, North Lanarkshire, Scotland). The cells were trypsinized, rinsed with PBS and then resuspended in culture medium without supplements. Each sample (1×10^6^ cells) was analyzed during incubation in a magnetically stirred chamber over a period of 5 min at 37°C. The signals were detected and analyzed using software from StrathKelvin Instruments. The rate of oxygen consumption was normalized to the number of living cells.

### Cytochrome oxidase activity assay

The activity of cytochrome oxidase in IMQ-treated and untreated cells was analyzed by Cytochrome Oxidase Activity Colorimetric Assay Kit (BioVision) that was performed according to manufacturer's instruction. In short, the cells were maintained in 6-well plates and treated with or without IMQ. After the indicated time points, total cell lysate were harvested. For each reaction, the 30 μg cell lysate was mixed with 120 μl of reduced cytohrome c. We measured the reduction in absorption at the wavelength of 550 nm using UV/Vis UV-1700 PharmaSpec spectrophotometer (Shimadzu, Benelux BV, Hertogenbosch, The Netherlands) over a period of 30 ~ 45 minutes. The reduced absorbance was normalized to the amount of protein and the difference in time. The reduced absorbance of untreated group was as 1 fold.

### Mitochondrial potential assay

The influence of mitochondrial respiration in IMQ-treated and untreated cells was determined by MitoTracker Red CMXRos (Invitrogen, Carlsbad, CA, USA) using flow cytometry. In brief, the cells were maintained in 6-well plates with or without 50 μg/ml IMQ treatment. After the indicated time points, collected cells and stained by MitoTracker Red CMXRos for 5 minutes, cells were washed by PBS then maintained in completed culture medium. The red fluorescence of MitoTracker Red CMXRos was detected by flow cytometry with FL2 channel. Analyzed the mitochondrial potential by overlay histogram plots of between IMQ-treated and untreated control.

### Reverse transcriptase-PCR and real-time PCR

Total RNA was isolated using TRIzol reagent. First-strand cDNA was synthesized from total RNA using the Sprint PowerScript PrePrimed Single Shots Kit (Clontech, Mountain View, CA, USA). PCRs were performed using the cDNAs (35 cycles at 94°C for 30 seconds, 60°C for 30 seconds and 68°C for 1 minute) using Titanium Taq DNA polymerase (Clontech). The primer pairs were as follows: human TLR7 forward, 5'-GGGCCCATCTCAAGCTGATC-3', and reverse, 5'- TGTGAAAGGACGCTGGGGAG-3'; human TLR8 forward, 5'- TGCGCTGCTGCAAGTTACGG-3', and reverse, 5'- GTTGAGGAATGCCCCGTCTG-3'; and β-actin forward, 5'-ATTGCCGACAGGATGCAGAA-3', and reverse, 5'-GCTGATCCACATCTGCTGGAA-3'. All PCR products were fractionated by 2% agarose gel electrophoresis and visualized with ethidium bromide. For real-time PCR analysis, the synthesized cDNAs were mixed with 2X SYBR Green PCR Master Mix (Applied Biosystems, Foster City, CA, USA), a pair of gene-specific forward and reverse primers (HIF-1α: 5'-TATGAGCCAGAAGAACTTTTAGGC-3', 5'-GATGGCAGTAGCTGCGCTGATA-3'; VEGF: 5'-TTGCCTTGCTGCTCTACC TCCA-3', 5'-GATGGCAGTAGCTGCGCTGATA-3'; GLUT1: 5'-TTGCAGGCTT CTCCAACTGGAC-3', 5'-CAGAACCAGGAGCACAGTGAAG-3') and subjected to real-time PCR quantification using an ABI 7300 Real-Time PCR System (Applied Biosystems). All reactions were performed in quadruplet. The relative amounts of mRNAs were calculated using the comparative CT method. Human GAPDH mRNA was used as the internal control (forward primer: 5'-ACCACAGTCCATGCCATCAC-3'; reverse primer: 5'-TCCACCACCCTGTTGCTGT-3').

### Luciferase reporter assay

Cells were co-transfected with an HRE promoter reporter plasmid (pHIF1 luciferase reporter, SABiosciences, Valencia, CA) or a STAT3 promoter reporter plasmid (pSTAT3 luciferase reporter, SABiosciences) and a control Renilla luciferase reporter plasmid (Promega, Madison, WI). At 24 hours after transfection, the cells were treated with IMQ and then harvested. Luciferase activity was measured with a dual-luciferase reporter assay system (Promega). Light units were normalized to the Renilla luciferase activity.

### Transient transfection

A human HIF-1α small interfering RNA (siRNA; Santa Cruz) or pCMV1-Flag plasmids encoding human TLR7 and TLR8 were transiently transfected into cells using the Lipofectamine 2000 transfection reagent according to the manufacturer's instructions (Invitrogen). After indicated time, the cells were treated with IMQ for the indicated time and subjected to other assays.

### DNA content assay

Cells were incubated in normal culture medium or medium supplemented with 2-DG or 17-AAG or chetomin, with or without IMQ. At the indicated time, the cells were harvested and fixed in 70% ethanol at 4°C overnight. After centrifugation, the cell pellets were washed and resuspended in 0.6 ml of PBS with PI (40 μg/ml), and then, the cells were stained at 37°C for 30 min. After staining, the cells were centrifuged at 1200 rpm for 5 min. The supernatant was discarded, and the pellet was resuspended in PBS. The fluorescence emitted by the PI-DNA complex was quantified after laser excitation of the fluorescent dye using a Cytomics™ FC500 Flow Cytometer (Beckman Coulter, Fullerton, CA, USA).

### Immunoblotting

Cells were harvested by centrifugation, and washed with PBS. Whole- cell lysates were resuspended in PRO-PREP protein extraction solution (iNtRON, Taipei, Taiwan) containing a protease inhibitor cocktail. The extracts were vigorously shaken at 4°C for 15 min, followed by centrifugation. The supernatants were collected, and the protein concentrations were determined using the Bio-Rad BCA reagent (Bio-Rad Hercules, CA, USA). A 30 μg sample of each lysate was subjected to electrophoresis on SDS-polyacrylamide gels. The samples were electroblotted onto PVDF membranes. After blocking, the membranes were incubated with primary antibodies in TBST at 4°C overnight. They were then washed four times and incubated with horseradish peroxidase (HRP)-conjugated goat anti-mouse or rabbit IgG (Upstate, Billerica, MA, USA) for 2 hours. After washing with TBST, the blots were incubated for 1 min with the SuperSignal West Pico ECL reagent (Pierce Biotechnology, Rockford, IL, USA), and chemiluminescence was detected using by exposure to Kodak-X-Omat film. β-actin signals were used to verify equal protein loading in each lane.

### Immunocytochemistry

Cells were grown overnight on cover slides and treated with IMQ for the indicated time at 37°C. The cells were fixed in 1% paraformaldehyde at 4°C for 2 hours, washed with PBS and permeabilized by 0.1% Triton X-100/PBS containing 0.5% BSA for 30 min. For intracellular staining, the cells were blocked with 2% normal horse serum, incubated with antibodies against HIF-1α in PBS containing 0.05% Tween-20 (PBST) and then incubated with a FITC-conjugated goat anti-rabbit IgG (Sigma). After washing with PBST, the cells were mounted in a water-based mounting medium containing an anti-fade agent and DAPI (Vector Lab, Burlingame, CA, USA) and analyzed by confocal microscopy (Olympus, FV1000D, Tokyo, Japan).

### Mouse xenograft model

B16F10 cells were cultured with DMEM supplemented with 10% FBS. At approximately 70~80% confluence, the cells were harvested, washed and then resuspended in PBS containing matrix gel at 2×10^6^ cells/ml. Cells (2 × 10^5^) were injected subcutaneously into mice, which were monitored for tumor formation. When the tumor length or width reached 0.2 mm^2^, the tumors were injected with PBS or 2-DG (62.5 mg/kg) (or 17-AAG at 40 mg/kg, chetomin at 1 mg/kg) with or without IMQ (25 mg/kg) once per day. The tumor was determined daily by measuring the length and width. Tumor volume was calculated as: volume = (length × width^2^ × π)/6. After 8 days, the mice were sacrificed, and the tumors were harvested. The tumor weights were measured, and images were taken. All animal care and experimental procedures were approved and conducted by the Committee for Animal Experiments, National Chung Hsing University, Taichung, Taiwan (approved document NCHU-101-89).

### Statistical analyses

Three independent experiments were conducted in all studies, and all assay conditions were performed in duplicate or triplicate. The data were analyzed using Student's *t*test, and differences were considered significant when *p* values < 0.05.

## SUPPLEMENTARY FIGURES


