# Supplementary material for: Targeting Aerobic Glycolysis and HIF-1α Expression Enhance Imiquimod-induced Apoptosis in Cancer Cells
Source: Oncotarget. 2014 Mar 12;5(5):1363–81. doi: 10.18632/oncotarget.1734 (PMC4012728; doi:10.18632/oncotarget.1734)
Supplement: Supplementary file 1 [file oncotarget-05-1363-s001.pdf]

## SUPPLEMENTARY DATA

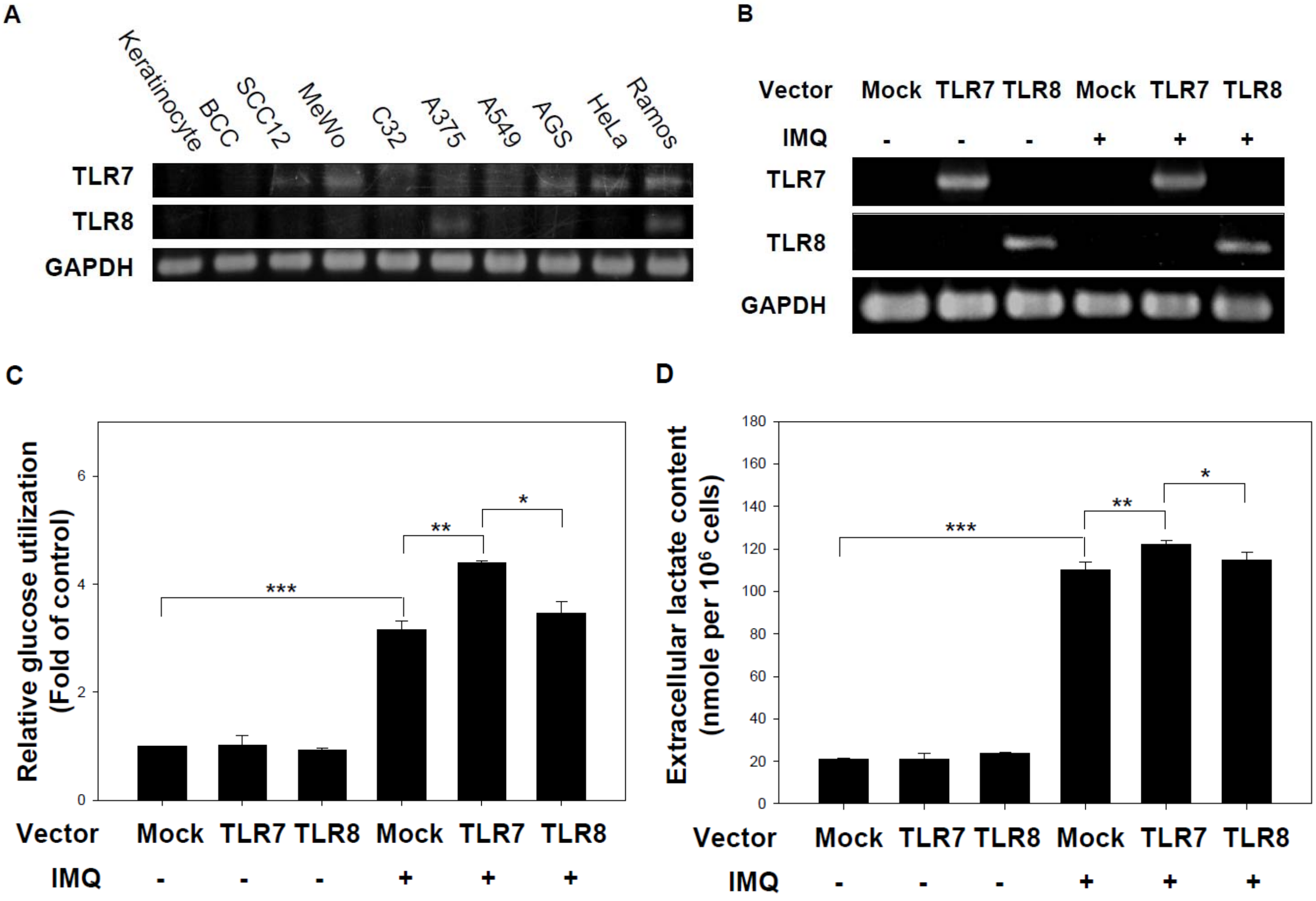


**Figure S1: IMQ-enhanced aerobic glycolysis may occur in TLR7/8 non-expressed tumor cells and the expression of TLR7 may strengthen the IMQ-enhanced effects.** **(A)** The mRNA expression patterns of TLR7 and TLR8 in various tumor cell lines and primary human keratinocytes. RT-PCR was used to analyze the mRNA expression of TLR7 and TLR8 in BCC, A549, A375, AGS, HeLa, C32, MeWo and SCC12 cells and keratinocytes. The Ramos B cell line was used as a TLR7/8 expressed control. **(B)** Over-expression of TLR7 and TLR8 in BCC cells. The BCC cells were transiently transfected with pCMV1-Flag-TLR7 or pCMV1-Flag-TLR8. After 48 hr, the mRNA expression of TLR7 and TLR8 were analyzed by RT-PCR. The pCMV1-Flag vector was used as a mock control. **(C and D)** Over-expression of TLR7 but not TLR8 could further enhance the IMQ-enhanced aerobic glycolysis in transfected BCC cells. After 48 hr, the TLR7 and TLR8 transfected BCC cells were incubated in culture medium with or without 50 µg/ml IMQ for 12 and 24 hours, and then, the relative glucose utilization **(C)** and lactate content **(D)** were analyzed using a glucose content assay and a lactate content assay with normalization based on the total cell number, respectively. The data are expressed as the mean ± S.E.M. of at least three independent experiments. * $p<0.05$; ** $p<0.01$.

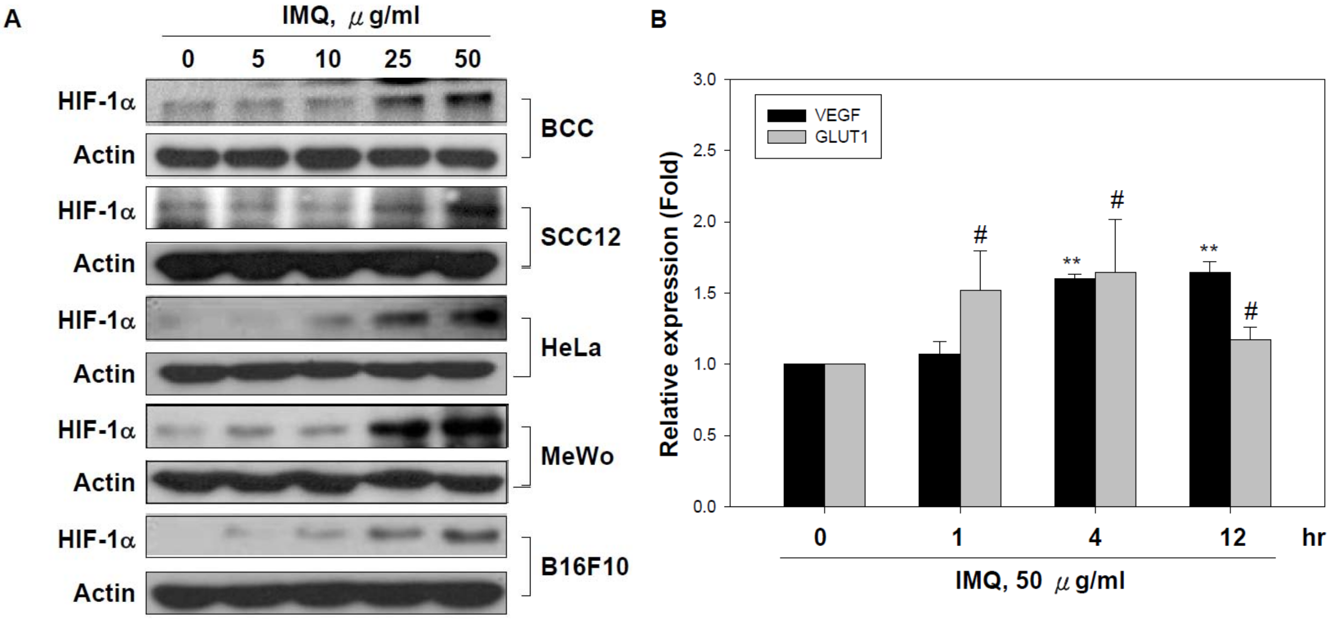


**Figure S2: IMQ induced HIF-1α expression in a dose-dependent manner and activated HIF-1α target genes in BCC and SCC12 cells.** **(A)** BCC, SCC12, MeWo, B16F10 and HeLa cells were treated with 0, 5, 10, 25 or 50 μg/ml IMQ for 4 hours, and cell lysates were then prepared for immunoblotting with HIF-1α and β-actin antibodies. **(B)** BCC and SCC12 cells were incubated in medium containing 50 μg/ml IMQ for 0, 1, 4 or 12 hours and then analyzed by quantitative real-time PCR to determine the mRNA levels of VEGF, GLUT1 and GAPDH. The data are expressed as the mean ± S.E.M. of at least three independent experiments. *, # $p<0.05$; ** $p<0.01$.

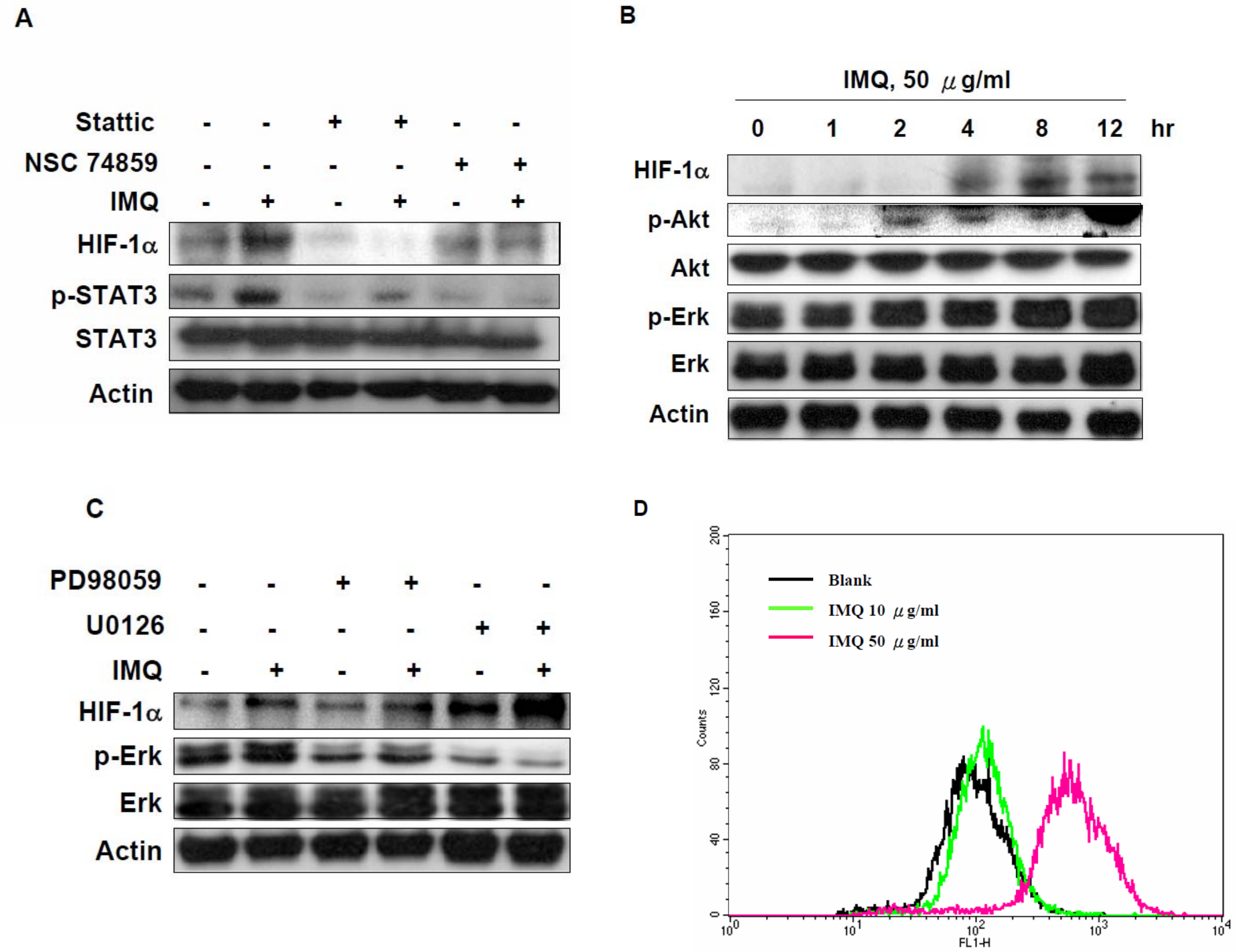


**Figure S3: The IMQ-induced increase in HIF-1α expression was not correlated with the activation of Erk in tumor cells.** **(A)** The pharmacological inhibition of STAT3 reduced IMQ-induced HIF-1α expression in SCC12 cells. SCC12 cells were pre-treated with 10 μM Stattic or 1 μM NSC74859 for 1 hour and then stimulated with 50 μg/ml IMQ for 4 hours. The cell lysates were collected for immunoblotting with HIF-1α, p-STAT3, STAT3 and β-actin antibodies. **(B)** IMQ-induced HIF-1α expression was associated with Akt and Erk activation in SCC12 cells. SCC12 cells were treated with 50 μg/ml IMQ for 0, 1, 2, 4, 8 or 12 hours then processed for immunoblotting to analyze the levels of HIF-1α, p-Akt, Akt, p-Erk, Erk and β-actin using specific antibodies. **(C)** Inhibition of the Erk signaling pathway had no significant effect on IMQ-induced HIF-1α expression in BCC cells. BCC cells were treated with 20 μM PD98059 or 10 μM U0126 for 1 hour and then incubated with 50 μg/ml IMQ. After 4 hours, cell lysates were collected for immunoblotting with HIF-1α, p-Erk, Erk and β-actin antibodies. **(D)** IMQ induced ROS production in BCC cells. BCC cells were treated with IMQ (10 or 50 μg/ml) for 2 hours. The cells were harvested and incubated with 10 μM DCFDA for 15 minutes and then analyzed by flow cytometry.

(*Continued*)

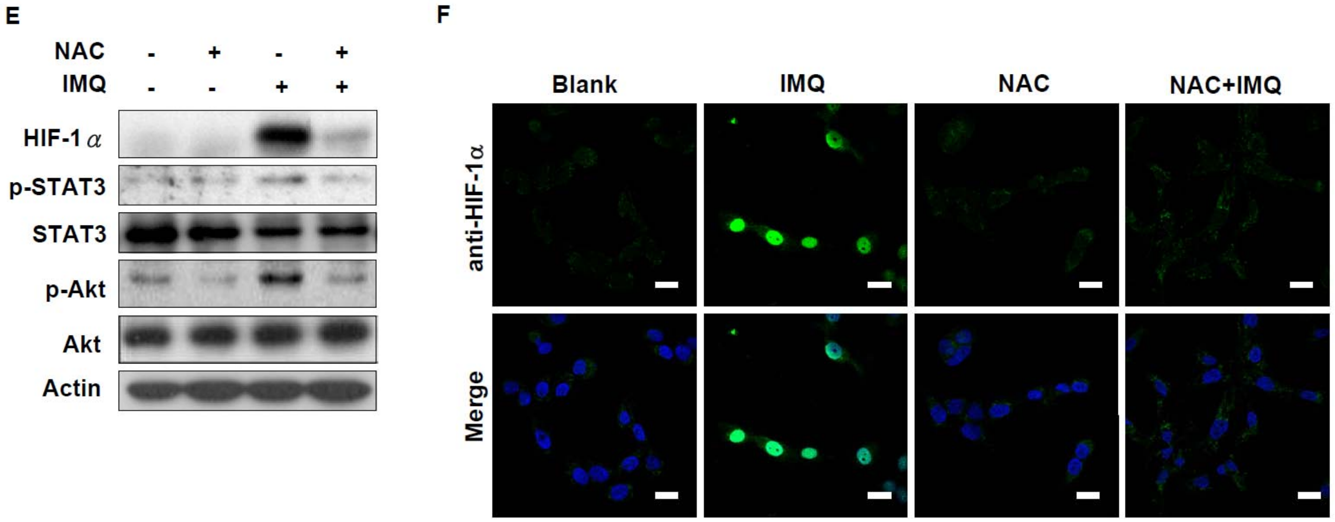


**Figure S3:** **(E** and **F)** The depletion of ROS reduced IMQ-induced HIF-1α expression, HIF-1α nuclear translocation, STAT3 phosphorylation, and decreased Akt activation in SCC12 cells. SCC12 cells were pre-treated with 2 mM NAC for 30 minutes and then treated with 50 μg/ml IMQ for 4 hours. Cell lysates were then collected for immunoblotting to detect the HIF-1α, p-Akt, Akt, p-STAT3, STAT3 and β-actin levels using antibodies **(E)** or for immunocytochemistry to determine the subcellular localization of HIF-1α using anti-HIF-1α antibodies **(F)**. Scale bars, 20 μm.

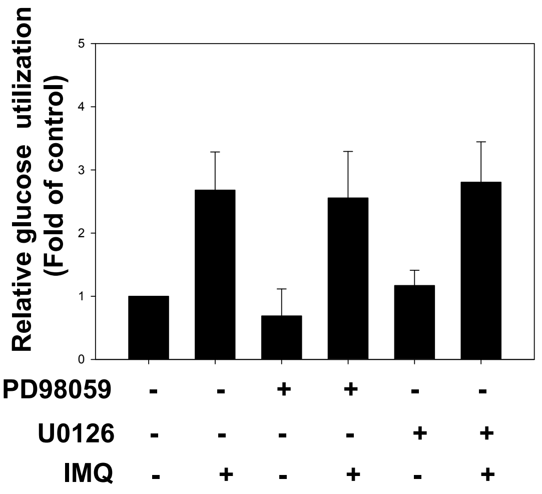


**Figure S4: The pharmacological inhibition of Erk did not influence IMQ-induced glucose utilization in BCC cells.** BCC cells were pre-treated with 20 µM PD98059 or 10 µM U0126 for 1 hour and then treated with 50 µg/ml IMQ for 12 hours. The cells were then processed for the glucose content assay. The fold change in relative glucose uptake was normalized to the total cell number.

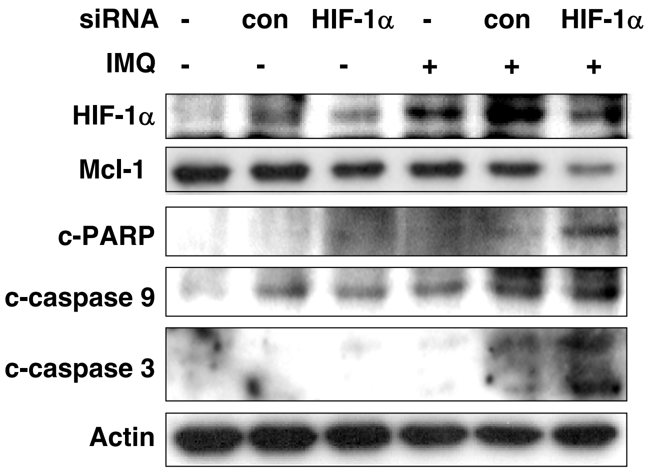


**Figure S5: HIF-1α expression mediated IMQ-induced apoptosis in SCC12 cells.** SCC12 cells were transfected with a HIF-1α siRNA or a non-specific control siRNA for 24 hours and then treated with 50 μg/ml IMQ for 24 hours. Cell lysates were collected and analyzed by immunoblotting with antibodies against cleaved-PARP, cleaved-caspase 9, cleaved-caspase 3, Mcl-1 and β-actin. The data are expressed as the mean ± S.E.M. of at least three independent experiments. * $p<0.05$; ** $p<0.01$.

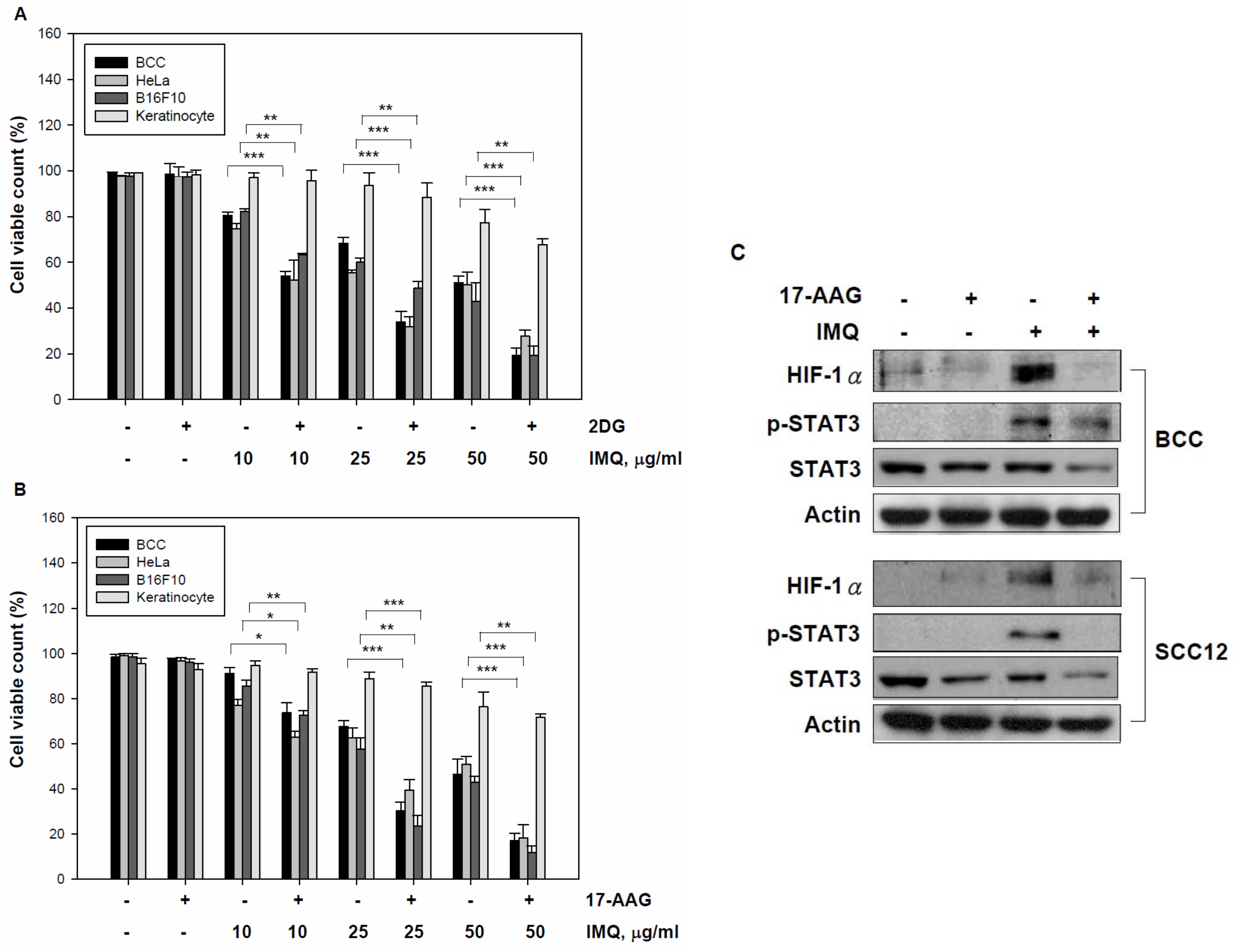


**Figure S6: 2-DG and 17-AAG synergized with IMQ to decrease the viability of tumor cells but not primary human keratinocytes. (A)** 2-DG enhanced the ability of IMQ to decrease the viability of BCC, HeLa and B16F10 cells but not primary human keratinocytes. **(B)** Combination treatment with IMQ and 17-AAG decreased the cell viability to a greater extent than either single agent alone in BCC, HeLa and B16F10 cells but not in primary human keratinocytes. Cells were treated with IMQ (0, 10, 25, 50 µg/ml) with or without 2-DG (50 mM for BCC and primary keratinocyte; 5 mM for HeLa and B16F10) or 17-AAG (10 µM) for 24 hours, and cell viability was then determined by counting viable cells after trypan blue staining. **(C)** Combination treatment with IMQ and 17-AAG inhibited STAT3 phosphorylation and HIF-1α protein expression. Cells were treated with IMQ (50 µg/ml) with or without 17-AAG (10 µM) for 24 hours. Protein expression was determined by immunoblotting. The data are expressed as the mean ± S.E.M. of at least three independent experiments. * $p<0.05$; ** $p<0.01$; *** $p<0.001$.

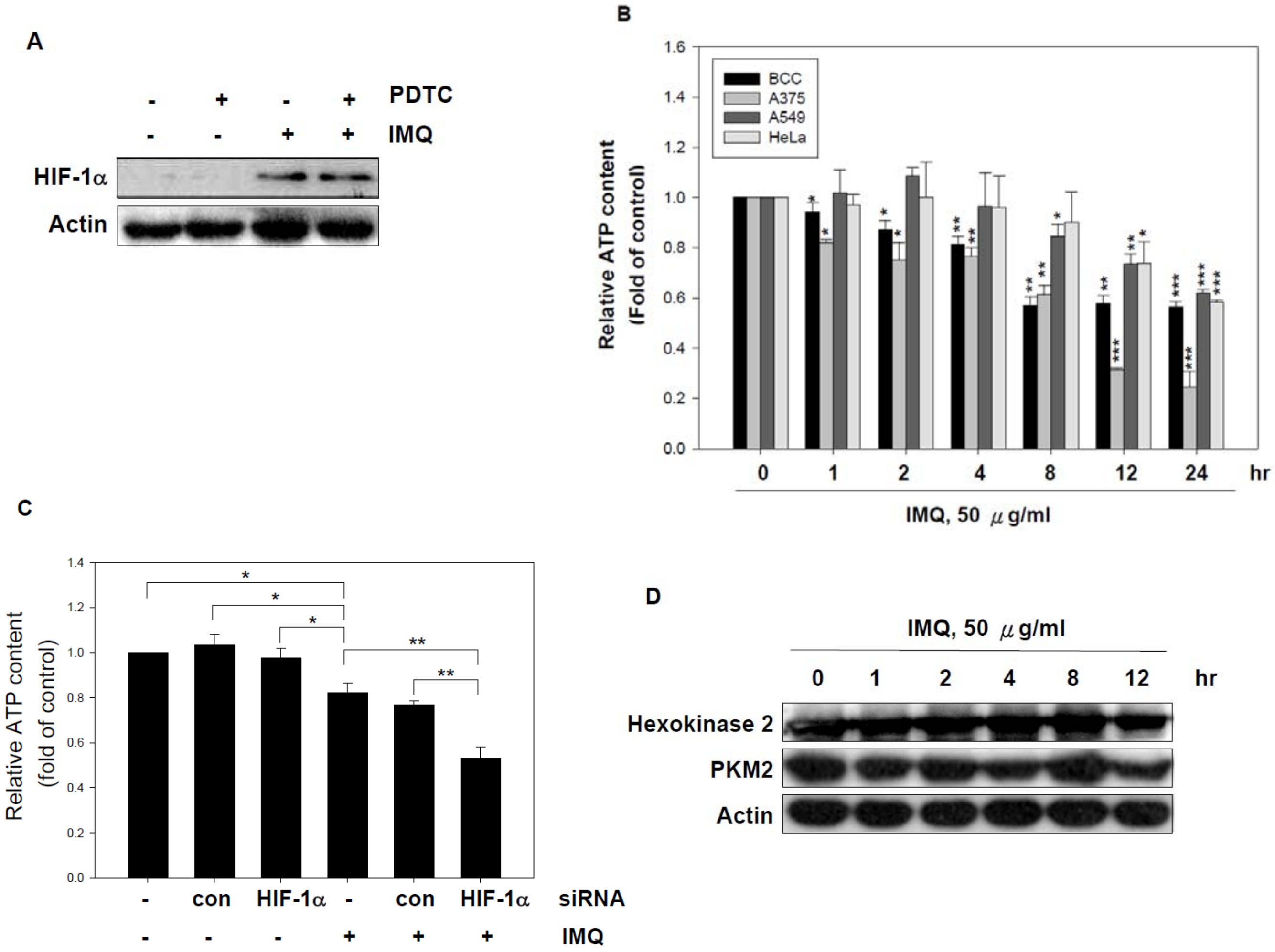


**Figure S7: Evaluation of the involvement of other pathways and ATP depletion in IMQ-induced aerobic glycolysis.** **(A)** NF-κB was not involved in IMQ-induced HIF-1α expression in BCC cells. BCC cells were pre-treated with the pharmacological NF-κB inhibitor PDTC (25 μM) for 1 hour and then treated with IMQ (50 μg/ml) for 4 hours. The cells were collected for immunoblotting with HIF-1α and β-actin antibodies. **(B** and **C)** IMQ depleted the intracellular ATP level in tumor cells. BCC, A375, A549 and HeLa cells were treated with IMQ 50 μg/ml for 0, 1, 2, 4, 8, 12 and 24 hours and then harvested to determine the intracellular ATP content **(B)**. **(C)** BCC cells were transfected with a HIF-1α siRNA or a control siRNA for 24 hours and then treated with IMQ (50 μg/ml) for 4 hours. The cells were collected and then processed for the intracellular ATP content assay. The fold change in the intracellular ATP level was normalized to the total cell number, and the intracellular ATP level of the untreated group was used as the baseline value to calculate the fold change. **(D)** The expression of PKM2 and hexokinase 2 did not be induced in IMQ-treated BCC cells. BCC cells were treated with IMQ (50 μg/ml) for 0, 1, 2, 4, 8 or 12 hours, and cell lysates were then prepared for immunoblotting with PKM2, hexokinase 2 and β-actin antibodies. The data are expressed as the mean ± S.E.M. of at least three independent experiments. * $p<0.05$; ** $p<0.01$; *** $p<0.001$.

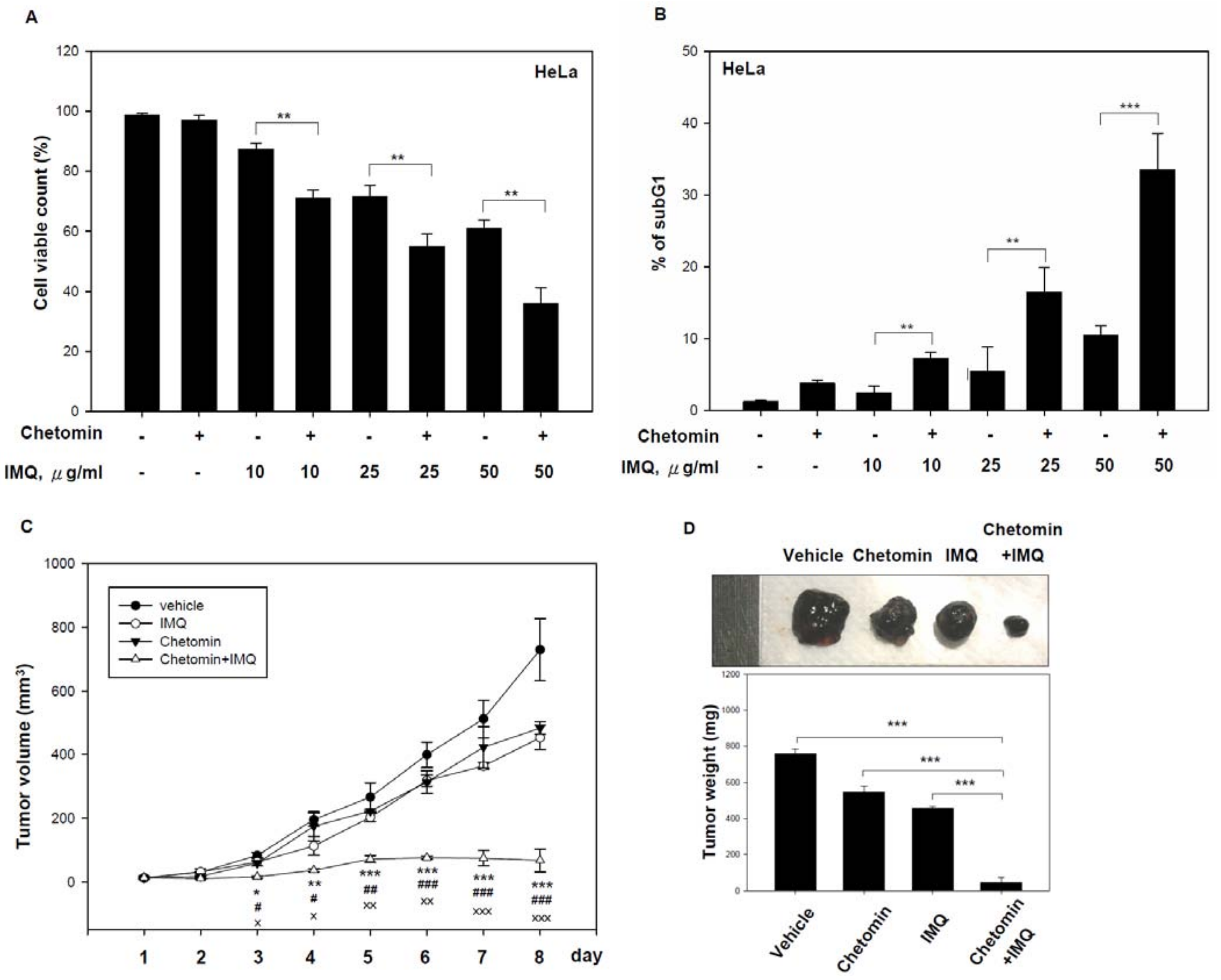


**Figure S8: The combination of a HIF-1α inhibitor chetomin with IMQ significantly suppressed tumor growth *in vitro* and *in vivo*.** (**A** and **B**) The HIF-1α inhibitor chetomin enhanced the IMQ induced cell death **(A)** and apoptosis **(B)** in HeLa cells. HeLa cells were treated with IMQ (0, 10, 25, 50 µg/ml) with or without chetomin (200 nM) for 24 hours. The cell viability and apoptosis were then determined by using cell viable counting and DNA content assay, respectively. **(C and D)** The chetomin synergized with IMQ to suppress tumor growth *in vivo*. B16F10 melanoma-bearing mice were generated as previously described and treated daily with 1 mg/kg chetomin, 25 mg/kg IMQ or a combination of chetomin and IMQ by *in situ* injection at the tumor site. The tumor volume was measured daily for one week **(C)**. All the animals were sacrificed after 8 days of treatment, and the tumor weights were recorded **(D)**. The asterisk represents significance between control and chetomin+IMQ (C). The pound represents significance between IMQ and chetomin +IMQ (C); the double dagger represents significance between chetomin and chetomin +IMQ (C). The data are expressed as the mean ± S.E.M. of at least three independent experiments (* $p<0.05$; ** $p<0.01$; *** $p<0.001$; # $p<0.05$; ## $p<0.01$; ### $p<0.001$; × $p<0.05$; ×× $p<0.01$).
